# Hyperradiance by a stream of phase-correlated atomic dipole pairs traversing a high-Q cavity

**DOI:** 10.1038/s41598-021-90669-7

**Published:** 2021-05-27

**Authors:** Junseok Han, Jinuk Kim, Seung-hoon Oh, Gibeom Son, Junseo Ha, Kyungwon An

**Affiliations:** Department of Physics and Astronomy & Institute of Applied Physics, Seoul National University, Seoul, 08826 Korea

**Keywords:** Quantum optics, Atomic and molecular interactions with photons

## Abstract

Hyperradiance in which radiation rate exceeds that of superradiance has been theoretically investigated in various coherently-coupled emitter-field systems. In most cases, either proposed setups were experimentally challenging or the mean photon number in a cavity was limited. In this paper, with numerical simulations and analytic calculations, we demonstrate that significant hyperradiance with a large mean photon number can occur in a microlaser system, where pairs of two-level atoms prepared in quantum superposition states traverse a high-*Q* cavity in the presence of a pump field intersecting the cavity mode. Hyperradiance is induced when the intracavity-pump Rabi frequency is out of phase with respect to the atom-cavity coupling so that the reduction of atomic polarization by the atom-cavity coupling is compensated by the pump Rabi frequency in the steady state to maximize atomic photoemission.

## Introduction

Superradiance, first proposed by R. Dicke, refers to a quantum mechanical emission phenomenon where a special entangled state or Dicke state of *N* correlated atoms exhibits emission power proportional to $N^2$^1^, in contrast to ordinary spontaneous as well as stimulated emission just proportional to *N*^2^. Many studies have performed theoretical investigation of superradiance^3–7^ as well as experimental realization in various systems^8–13^. There are also studies to manipulate and engineer superradiance in order to boost superradiance behavior itself^14–17^. Superradiance as a cooperative emission process due to quantum correlation, is often considered to provide the highest emission enhancement that the quantum correlation can offer.

However, in recent years, several authors pointed out that under proper conditions the emission rate can exceed that of superradiance. Emission in such circumstances is called hyperradiance and it has been theoretically studied in various physical systems such as coupled linear Josephson junctions^18–21^ and atom-cavity systems^22–24^. In the former, the coupling between soliton oscillators can make the microwave emission exceeds that of superradiance. In the latter,  Pleinert et al. considered an atom-cavity system driven by a pump laser while the spatial phase of the atoms inside the cavity is manipulated to suppress the destructive interference between different transition processes and to obtain the emitted number of photons surpassing that of superradiance^22^.  Xu et al. and  Zhao et al. considered a similar atom-cavity system but introduced frequency detuning among the cavity, the atoms and a pump laser^23,24^. Their studies showed that nonzero atom-cavity or cavity-pump detuning can bring about hyperradiance, contrary to the usual expectation that the emission be the strongest on resonance.

These studies on hyperraidance are significant advancements showing that the quantum correlation can be manipulated to yield further enhancement beyond the superradiance’s N-squared dependence. However, the hyperradiance studies so far have focused mostly on the increased emission rate, quoting very low mean photon numbers in the cavity or not properly addressing available emission power.

In this paper, we propose a different approach to induce hyperradiance in the setting of a microlaser^25^, where a stream of coherent atomic dipole pairs traverses a high-*Q* cavity. Coherent single-atom superradiance as well as superabsorption have been experimentally realized in a similar setup^26,27^. It is thus natural to consider a possibility of hyperradiance in the microlaser. With numerical simulations and analytic calculations, we demonstrate that significant hyperradiance can occur when a pump field intersecting the cavity mode is introduced with opposite phase to the atom-cavity coupling. Under this condition, the pump Rabi frequency counteracts the atom-cavity coupling and thus keeps the atomic polarization maximized during the atom-cavity interaction time, resulting in enhanced photoemission. We also discuss the feasibility of realizing hyperradiance experimentally in the microlaser. Our microlaser setup is experimentally easier to access than the setups considered in the previous theoretical studies, namely a pair of atoms continuously trapped exactly at antinodes in a cavity. We confirm that the present scheme is robust to random fluctuations such as random arrival times of atoms and atomic velocity distributions, which inevitably occur in actual experiments. With experimentally achievable parameters we observe a hyperradiance behavior 8.5 times stronger than superradiance for one pair of atoms on average in the cavity, resulting in an average photon number in the cavity up to $\langle \hat{n} \rangle \sim 31$, corresponding to an output photon flux of $1.6\times 10^7$/s for a cavity decay rate of 82.5kHz. We expect that our study would serve as a stepping stone to future theoretical as well as experimental studies on hyperradiance and to the development of ultrahigh-efficient microlasers.

## Results

### Model system

**Figure 1:**
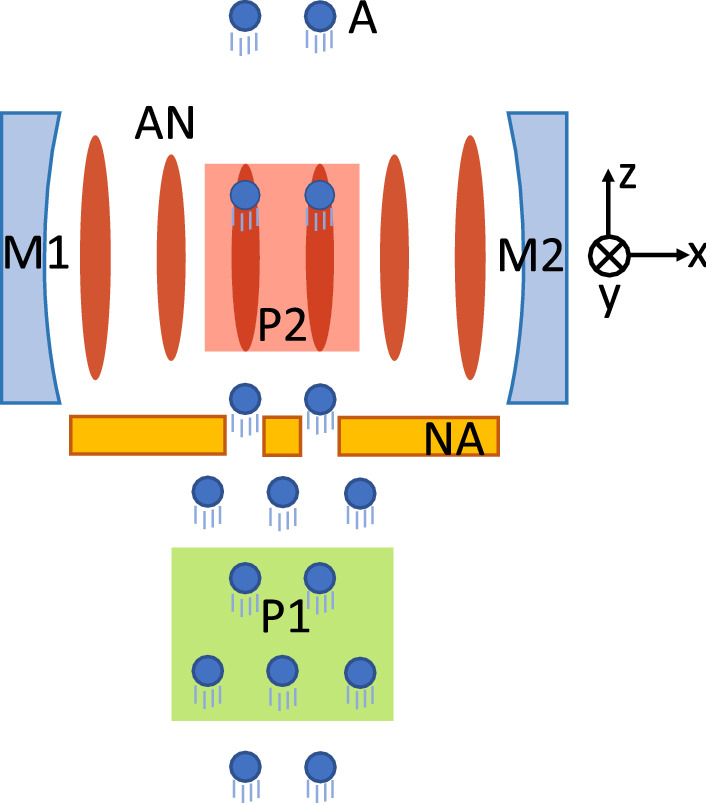
Our model system. M1, M2: cavity mirrors. A: a beam of atom pairs propagating in z direction. P1: the first pump laser (propagating in y direction) to prepare atoms in the superposition state. P2: the second pump beam (propagating in y direction) intersecting the cavity mode perpendicularly. AN: the anti nodes (denoted by red-filled ellipses) of the cavity field. NA: a nanohole array aperture allowing the atoms to move along the anti-nodes of the cavity field. The two pump lasers are resonant with the cavity, which is detuned from the atomic transition frequency in general.

Figure 1 depicts a model system for numerical studies. A beam of two-level atoms in a pair traverses a high-*Q* cavity mode. The atoms are excited to a superposition state of the lower and upper levels with equal probabilities by the first pump laser before they enter the cavity. For a time duration $\tau$, the atoms interact with the cavity field in the presence of the second pump laser, which goes through the cavity perpendicularly to both the cavity axis (*x* axis) and the atomic beam direction (*z* axis). The atoms go through the anti-nodes of the cavity mode so that they interact with the cavity field with the maximum coupling strength *g*. One way of achieving this arrangement is to employ a nanohole-array atomic beam aperture in front of the cavity^28^. After $\tau$, the atoms leave the cavity and simultaneously a new pair of atoms enter the cavity. This process is repeated until the cavity field reaches a steady state.

### Equations of motion

Let us denote the atomic transition frequency as $\omega _{\rm{a}}$, the cavity resonant frequency as $\omega _{\rm{c}}$, the frequency of the first pump lasers as $\omega _{\rm{p1}}$ and that of the second pump laser as $\omega _{\rm{p2}}$. We assume $\omega _{\rm{c}}=\omega _{\rm{p1}}=\omega _{\rm{p2}} \ne \omega _a$, so detuning $\Delta =\omega _a-\omega _c=\omega _a-\omega _{p1}=\omega _a-\omega _{p2}\ne 0$ in general. Because atoms interact with the off-resonant pump lasers, the initial atomic state entering the cavity at time *t* is given by $\frac{1}{\sqrt{2}}\left[ \vert {\rm g} \rangle +e^{-i\left( \omega _{p1} t+\phi \right) }\vert {\rm e} \rangle \right]$ with $\phi$ a constant phase introduced by the first pump laser. The cavity decay rate $\kappa$ is not neglected but the atomic decay rate $\gamma$ is assumed to be negligible compared to the other rates($\gamma \ll \kappa , g$). The Hamiltonian in the interaction picture can be written as^29^

1 $$\begin{aligned} \hat{H}\, = \, & {} \frac{1}{2}\hbar \Delta \sum ^N_{i=1}\hat{\sigma }_{z i} +\hbar g\sum ^N_{i=1}\left( \hat{a}^\dagger \hat{\sigma }^-_i +\hat{a}\hat{\sigma }^+_i\right) \nonumber \\&+\hbar \Omega \sum ^N_{i=1}\left( \hat{\sigma }^+_i+\hat{\sigma }^-_i \right) , \end{aligned}$$

where *N* is the number of atoms interacting with the cavity at the same time, $\Omega$ the Rabi frequency of the second pump laser, $\hat{a}$ ($\hat{a}^\dagger$) the annihilation (creation) operator for the cavity field, $\hat{\sigma }_{zi}$ the Pauli *z*-matrix and $\hat{\sigma }_i^+$ ($\hat{\sigma }_i^-$) the raising (lowering) operator for the *i*th two-level atom, so $\hat{\sigma }_{zi}=\vert \mathrm e \rangle _i\langle \mathrm e \vert _i-\vert \mathrm g \rangle _i\langle \mathrm g \vert _i$, $\hat{\sigma }^+_i=\vert \mathrm e \rangle _i\langle \mathrm g \vert _i$ and $\hat{\sigma }^-_i=\vert \mathrm g \rangle _i\langle \mathrm e \vert _i$. When $N=1$, we have ‘one-atom case’ in which only one atom interacts with the cavity at a time. We have ‘two-atoms case’ when $N=2$ with a pair of atoms interacting with the cavity at a time. The atomic initial state in the interaction picture becomes $\frac{1}{\sqrt{2}}\left( \vert \mathrm g \rangle +e^{-i\phi }\vert \mathrm e \rangle \right)$ in the rotating frame of the first pump frequency. We use the following master equation to calculate the time evolution of the atom-cavity state^30^.

2 $$\begin{aligned} \frac{d}{dt}\hat{\rho }&=-\frac{i}{\hbar }\left[ \hat{H},\hat{\rho }\right] +\mathcal {L}\hat{\rho }, \end{aligned}$$

3 $$\begin{aligned} \mathcal {L}\hat{\rho }&=\frac{\kappa }{2}\left( 2\hat{a}\hat{\rho } \hat{a}^\dagger -\hat{a}^\dagger \hat{a}\hat{\rho }-\hat{\rho } \hat{a}^\dagger \hat{a}\right) , \end{aligned}$$

We solve Eqs. (2) and (3) numerically during the interaction time $\tau$. We describe the atoms entering the cavity with a tensor product of the atomic density matrix and the cavity density matrix. When the atoms leave the cavity after $\tau$, we take tracing out the atomic density matrix. The whole process is repeated until a steady state is reached. The time evolution of the average photon number $\langle \hat{n} \rangle =\langle \hat{a}^\dagger \hat{a} \rangle$, proportional to the emission power, can be calculated this way.

Degree of hyperradiance is characterized by a normalized radiance witness *R* defined as^22–24^

4 $$\begin{aligned} R=\frac{\langle \hat{n} \rangle _2-2\langle \hat{n} \rangle _1}{2\langle \hat{n} \rangle _1}, \end{aligned}$$

where $\langle \hat{n} \rangle _1$ is the average photon number in the steady state for the one-atom case ($N=1$) and $\langle \hat{n} \rangle _2$ for the two-atom case ($N=2$). In the classical limit without quantum correlation, the two-atom case would generate emission power two times more than the one-atom case. In this limit, we expect $R=0$. However, in superradiance, we expect the emission power to scale with $N^2$. We thus expect $\langle \hat{n} \rangle _2=4\langle \hat{n} \rangle _1$, indicating $R=1$. In the same line of reasoning, we expect that $R>1$ would indicate hyperradiance exceeding superradiance.

When the mean photon number is much larger than unity and the interaction time between atoms and the cavity remains brief ($g\sqrt{n}\tau \ll 1$) in our consideration, we can apply a semiclassical analysis to obtain analytic expressions for the mean photon number^31,32^. The expectation values of quantum operators like $\hat{a}$, $\hat{\sigma }^-_i$ and $\hat{\sigma }_{zi}$ are approximated to be classical quantities $\alpha$, $\sigma$ and *r*, respectively. This way, we can construct a set of semiclassical equations, equivalent to the Maxwell–Schrödinger equations, as^35^

5a $$\begin{aligned} \dot{\alpha }+\frac{\kappa }{2} \alpha\, = \, & {} -g\sigma \end{aligned}$$

5b $$\begin{aligned} \dot{\sigma }+i\Delta \sigma\, = \, & {} -(g\alpha -\Omega ) r\end{aligned}$$

5c $$\begin{aligned} \dot{r}\, = \, & {} 4\mathrm{Re}\left[ (g\alpha ^*-\Omega ^*)\sigma \right] , \end{aligned}$$

from which we can derive the following rate equation with identification $n= |\alpha |^2$

6 $$\begin{aligned} \dot{n}+\kappa n\, = \, & {} \frac{Ng\sqrt{n}}{2\tau }\frac{\sin {2\left( g\sqrt{n}\cos {\Delta \tau }+\mathfrak {I}[\Omega e^{-i\phi }]\right) \tau }+\sin {\Delta \tau }}{\Delta +2g\sqrt{n}\cos {\Delta \tau }+2\mathfrak {I}[\Omega e^{-i\phi }]} \nonumber \\&+(\Delta \longleftrightarrow -\Delta ), \end{aligned}$$

where $N=$1 or 2 and the short-hand notation $(\Delta \longleftrightarrow -\Delta )$ represents a term same as the one above except for all $\Delta$’s replaced with $-\Delta$.

The steady state solution of this rate equation can be solved analytically for $\Delta =0$. For the initial atomic state $\frac{1}{\sqrt{2}}\left( \vert \mathrm g \rangle -i\vert \mathrm e \rangle \right)$ ($\phi =\pi /2$) corresponding to $\sigma (0)=-N/2$, we obtain

7 $$\begin{aligned} n\approx & {} \left[ \frac{-3+4N (\Omega /\kappa ) (g\tau )^2+\sqrt{9-24N (\Omega /\kappa )(g\tau )^2+24N^2 (g/\kappa )^2(g\tau )^2}}{4N(g/\kappa ) (g\tau )^2}\right] ^2 \nonumber \\\approx & {} \left( \frac{gN}{\kappa }\right) ^2\left[ 1 -\frac{2}{3} (\Omega \tau )^2+\frac{4}{3}N ( \Omega /\kappa )(g\tau )^2 -\frac{2}{3}N^2 (g/\kappa )^2 (g\tau )^2\right] ^2, \end{aligned}$$

where the series expansion is done up to the second order of $\beta \tau$, where $\beta$ stands for $g, \Omega , \Delta$, or $\kappa$ or the multiplicative combinations of them. The condition $g, \Omega , \Delta , \kappa <1/\tau$ is assumed for the expansion. Using this result, we can derive a formula describing the condition under which the radiance witness *R* is locally maximized for $\Delta =0$ as follows.

8 $$\begin{aligned} \frac{\Omega }{\kappa } = 3\left( \frac{g}{\kappa }\right) ^2. \end{aligned}$$

The detailed derivation of Eqs. (6)–(8) is presented in “Methods”.

Equation (7) clearly shows the terms responsible for hyperradiance, namely the higher order terms than $N^2$ terms. Without these higher-order terms, we just get superradiance with *n* being proportional to $N^2$. As long as the third term proportional to *N* (enhanced emission) in the second bracket in Eq. (7) is larger than the fourth term proportional to $N^2$ (reabsorption) in the same bracket, which is the case when $\Omega /\kappa >(N/2)(g/\kappa )^2$, the higher-order terms produce more photons than superradiance and thus we get hyperradiance. It is clear that hyperradiance is not possible if $\Omega =0$ at least in the semiclassical limit with $n\gg 1$ (significant mean photon number). More discussion on the physical origin of the higher-order terms will be given when we examine the dependence on the pump Rabi frequency below.

**Figure 2:**
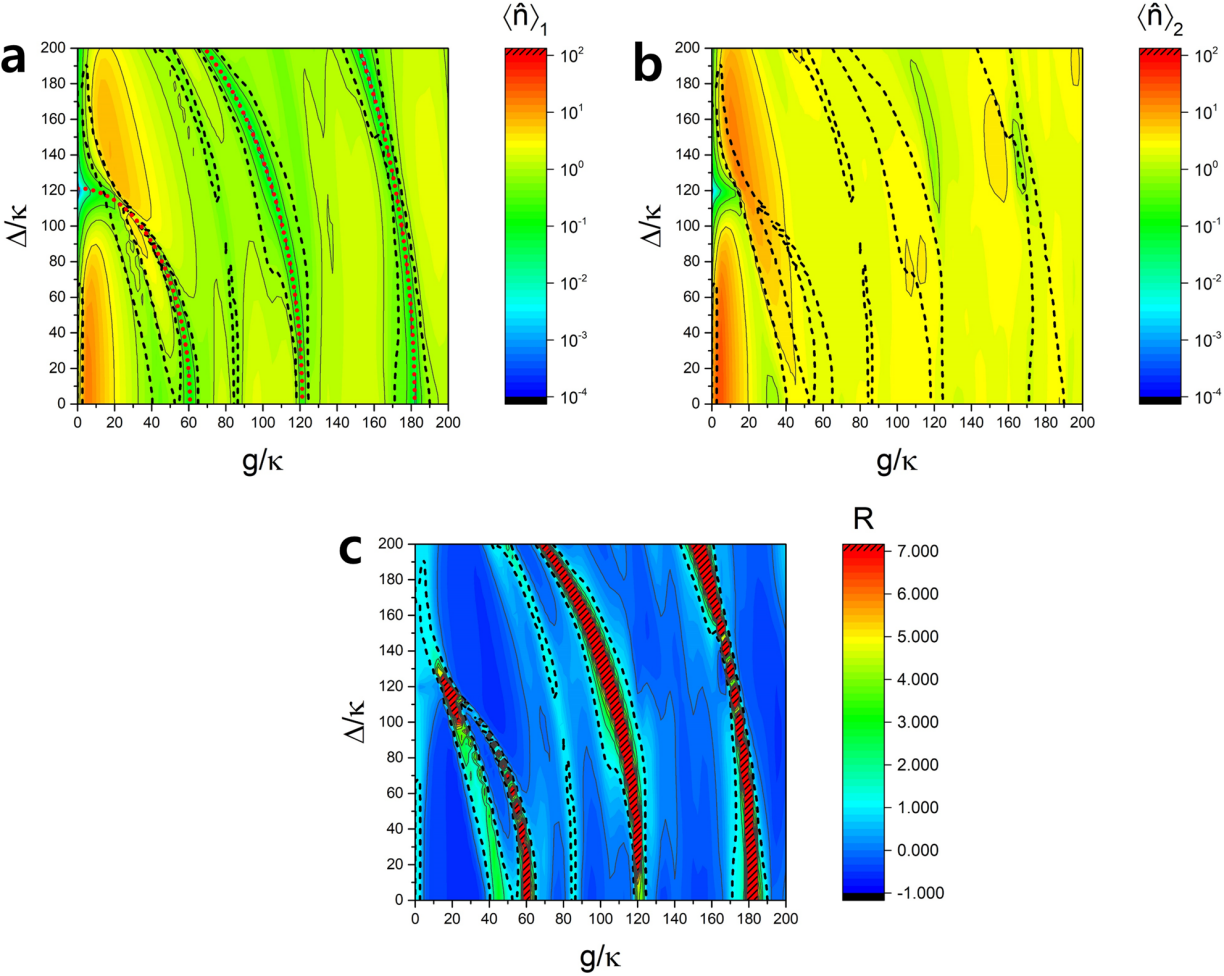
Dependance of hyperradiance on g/$\kappa$ and $\Delta /\kappa$. (**a**) Average number of photons in the cavity of one-atom case. Red dotted curves are the ellipsoidal curves given by Eq. (10). (**b**) The same for two-atom case. (**c**) Radiance witness *R*. The hatched red region indicates $R>7.0$. The Rabi frequency is $\Omega /\kappa =10$, the interaction time $\tau = 0.052/\kappa$, the cavity decay time $1/\kappa = 1.93$ $\mu$s and $\phi =\pi /2$. Hyperradiance region is inside the black dashed curves.

### Dependence on the atom-cavity detuning and the coupling for a fixed pump Rabi frequency

Numerical simulation results are summarized in Fig. 2, where the average photon numbers $\langle \hat{n} \rangle _1$ of one-atom case and $\langle \hat{n} \rangle _2$ of two-atom case as well as the consequent radiance witness *R* are plotted as functions of the detuning $\Delta$ and the coupling strength *g*. The regions of hyperradiance ($R>1$) are enclosed by the dashed dot lines. The hatched red regions correspond to $R>7$, where *R* can be as high as 296. We can see there are various hyperradiance regions. Majority of these hyperradiance regions lie on a set of ellipsoidal curves shown in Fig. 2a (red dotted curves). These ellipsoidal curves can be described as

9 $$\begin{aligned} \left( \sqrt{\left( \Delta /2\right) ^2+g^2}\right) \tau \approx m\pi \left( m=1,2,3\ldots \right) , \end{aligned}$$

or alternatively,

10 $$\begin{aligned} \left( \frac{\Delta /\kappa }{2}\right) ^2+\left( g/\kappa \right) ^2\approx \left( \frac{m\pi }{\kappa \tau }\right) ^2 \left( m=1,2,3\ldots \right) . \end{aligned}$$

What Eq. (9) or (10) means is as follows. First, note that the square-root expression in the parenthesis in Eq. (9) is the vacuum Rabi frequency in the presence of an atom-cavity detuning $\Delta$ (hereinafter the Rabi frequency in the presence of a detuning is referred to as ‘full Rabi frequency’). In one-atom case, for the atoms initially prepared on the equator in the Bloch sphere, a half or a full Rabi oscillation during the interaction time $\tau$ would then bring the Bloch vector of the exiting atoms to the equator again, resulting no net emission to the cavity. Since the emitted photons are re-absorbed by the atoms, the number of photons becomes relatively low in the one-atom case. We confirm that in Fig. 2a, the average photon number $\langle \hat{n} \rangle _1$ is much less than unity around the ellipsoidal curves. The approximation $g\sqrt{\langle \hat{n} \rangle _1+1}\approx g$ holds there and thus Eq. (9) describes a half or a full Rabi rotation in the region around the ellipsoidal curves. The Rabi frequency of the second pump laser $\Omega$ is not present in the above equations; its effect is reflected in the mean photon number $\langle \hat{n} \rangle _1$ of negligible magnitude.

The condition for suppression of the photon number is different for two-atoms case. Unlike the one-atom case, the full Rabi frequency of two-atom case is not simple. We even cannot apply the low photon number approximation because as we can check in Fig. 2b,c, the average photon number $\langle \hat{n} \rangle _2$ of two-atom case is comparable or larger than unity around the ellipsoidal curves. However, we can at least be sure that the full Rabi frequency of two-atom case is very different with that of one-atom case. The detailed arguments on the two-atom full Rabi frequency is presented in “Methods”.

The hyperradiance occurring around the elliptical curves given by Eq. (10) is of a quantum-mechanical origin (coherent Rabi oscillation), giving very low mean photon numbers and thus having little practical importance. Unlike the hyperradiance explained by the semiclassical theory of Eqs. (5), (6), and (7), requiring $\Omega >0$ at least, this type of hyperradiance can occur even when $\Omega =0$, as long as the condition by Eq. (10) is satisfied.

**Figure 3:**
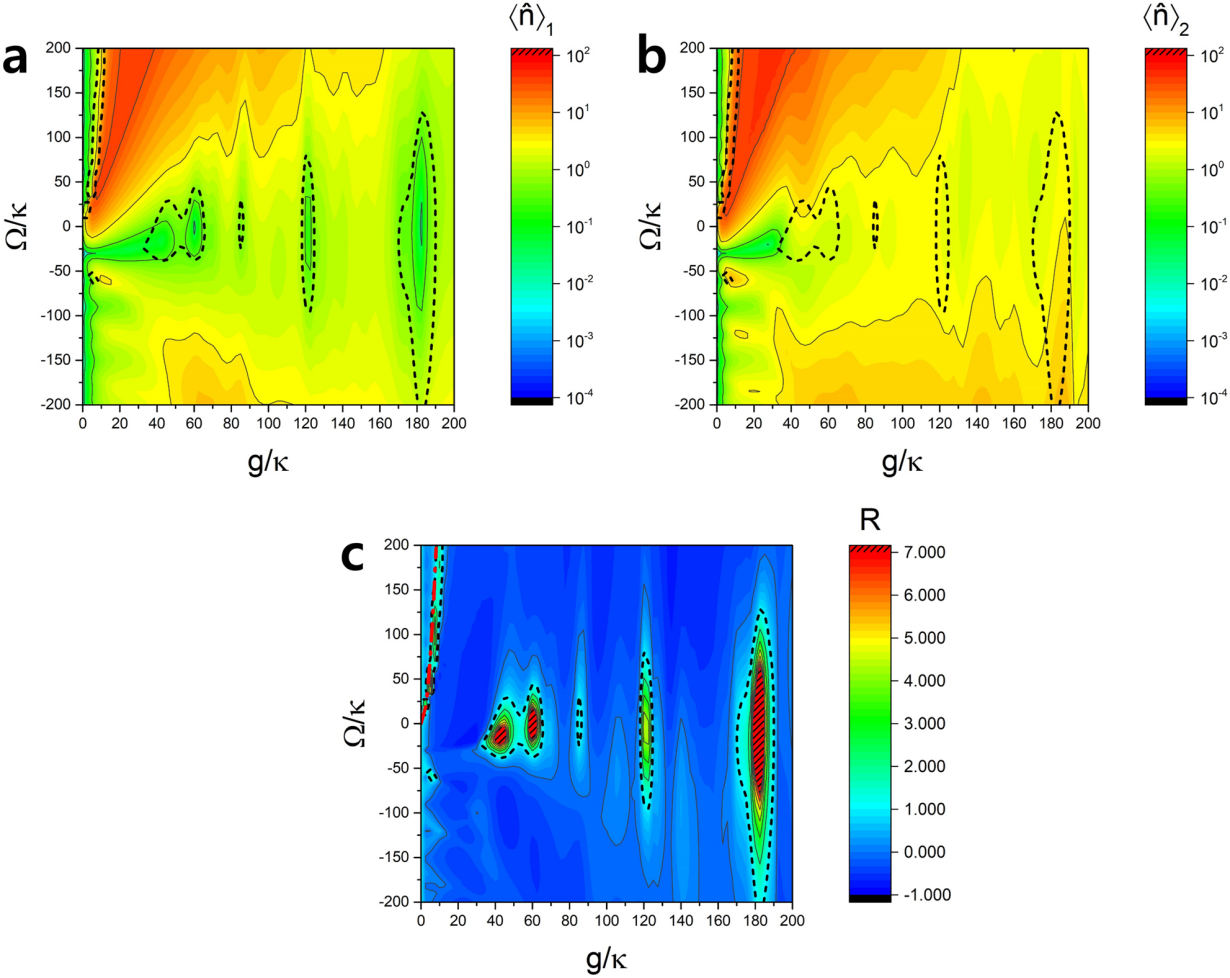
Dependance of hyperradiance on g/$\kappa$ and $\Omega /\kappa$. (**a**) Average number of photons in the cavity of one-atom case. (**b**) The same for two-atom case. (**c**) Radiance witness *R*. The hatched red regions indicates $R>7.0$. We consider a resonance case($\Delta =0$) with the interaction time $\tau = 0.052/\kappa$, the cavity decay time $1/\kappa = 1.93$ $\mu$s and $\phi =\pi /2$. The red dash-dot curve for small $g/\kappa$ is the plot of Eq. (8). Hyperradiance region is inside the black dashed curves.

### Dependence on the pump Rabi frequency and the coupling on resonance

It is noted that the mean photon numbers $\langle \hat{n} \rangle _1$ and $\langle \hat{n} \rangle _2$ are mostly significant for small *g* in Fig. 2. Moreover, they are maximized when $\Delta =0$. Since we are interested in large photon numbers, we rather consider $\Delta =0$ and vary $\Omega$ and *g* in this section. For the resonance case with $\Delta =0$, Fig. 3 depicts the same quantities as Fig. 2. The maximum radiance witness reaches up to approximately 100 in the hatched red region. There are hyperradiance regions with low *g* ($<10\kappa$) and with high *g* ($>30\kappa$). The low *g* region can be explained analytically using Eqs. (7) and (8) because they satisfy $g\tau <1$ condition. The red dash-dot line in Fig. 3c represents the curve given by Eq. (8). We can see that the numerical result agrees well with the analytic formula in the low *g* regions.

**Figure 4:**
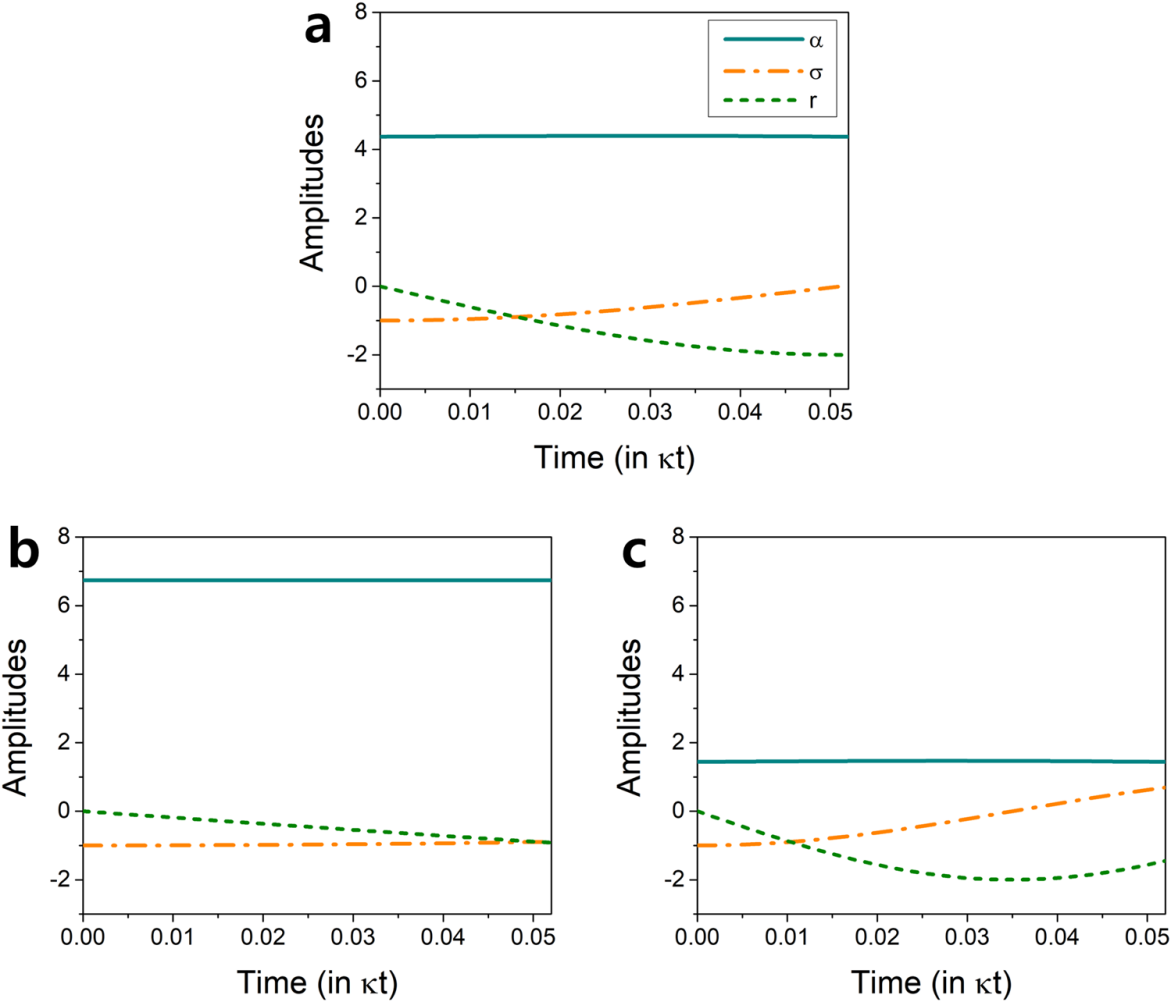
Origin of hyperradiance on resonance. (**a**) Evolution of $\alpha ,\sigma$ and *r* during the atom-cavity interaction time $\tau$ with $\kappa \tau =0.052$ when $\Omega =0,g/\kappa =3.5$ and $N=2$. The solution $\alpha$ corresponds to the steady-state solution of Eq. (5), satisfying $\alpha (0)=\alpha (\tau )$. (**b**) The same for $\Omega /\kappa =19$. This condition corresponds to the yellow points in Fig. 6. (**c**) The same for $\Omega /\kappa =-19$. For $\Omega >0$, the variation of $\sigma$ is minimized, staying close to its initial value and thus the atomic polarization is maximized, leading to a large $|\alpha |$. When $\Omega <0$, on the other hand, $\sigma$ varies more than that for $\Omega =0$, minimizing the polarization and making $|\alpha |$ stay low.

It is interesting that the mean photon numbers are much larger for $\Omega >0$ than $\Omega <0$. We can understand this as follows. When $\Omega >0$, the Rabi frequency of the second pump laser is out of phase with the vacuum Rabi frequency *g*. While the pump Rabi frequency tends to rotate the Bloch vector of the atoms upward, the vacuum Rabi frequency *g* tends to rotate it downward. As a result, atomic states can remain close to the equator during the interaction time and can produce more photons, which is then balanced by the cavity decay, resulting in a larger mean photon number. This behavior can be seen in Fig. 4 as well as in Eq. (5), where $\Omega >0$ ensures the slow change of $\sigma \sim \sigma (0)= -N/2$ (maximized polarization) and thus larger $|\alpha |$ in the steady state than the case with $\Omega =0$. Such maximized polarization during the interaction time makes the average photon number higher in low *g* region and even higher in the two-atom case, resulting in hyperradiance in the low *g* region. On the other hand, when $\Omega <0$, both the vacuum Rabi frequency and the pump Rabi frequency work together to rotate the Bloch vector downward faster, making the time-averaged value of $|\sigma |$ less and thus $|\alpha |$ smaller in the steady state than the case with $\Omega =0$. As a result we observe very low average photon numbers and no hyperradiance.

The hyperradiance in the low *g* region ($g<10\kappa$) for $\Delta =0$ has a potential to be utilized in various applications, such as in efficient light sources for lighting^33^ and visible light communication^34^, owing to its large mean photon number with a modest coupling constant easily achievable in experiments. Compared to other hyperradiance regions as well as the results in the previous studies, the low *g* region in the resonance case shows relatively large mean photon numbers in one-atom case, and even more in two-atom cases. We can obtain the mean photon number up to $\sim 40$ as can be seen in Fig. 6b, a magnified version of Fig. 3b.

There are much wider and bigger hyperradiance regions with high *g* around $g/\kappa \approx 60, 120,$ and 180. The average photon numbers in these regions are very low compared to the that of low *g* regions, but radiance witness *R* is much higher than that of low *g* regions regardless of the sign of $\Omega$. We can employ the similar argument we used for the off-resonance case in order to explain the hyperradiance in the high *g* regions. For $\Delta =0$, Eq. (9) is simplified as

11 $$\begin{aligned} g\tau \approx m\pi ,\left( m=1,2,3\ldots \right) \end{aligned}$$

or alternatively,

12 $$\begin{aligned} g/\kappa \approx \frac{m\pi }{\kappa \tau }\approx 60\times m, \left( m=1,2,3\ldots \right) \end{aligned}$$

for $\kappa \tau =0.052$ in Fig. 3, well accounting for the location of the high *g* regions of hyperradiance.

## Discussion

### Random injection case

So far, we have assumed an ideal situation where the atoms are injected into the cavity with an identical velocity at a regular time interval equal to $\tau$. We call this situation ‘regular injection case’. In real experiments, atomic velocities follow a distribution with a finite width. Moreover, atoms are injected into the cavity at random times while the average injection rate is constant. In order to account for such experimental randomness, we assume a Gaussian velocity distribution with a velocity deviation equal to 0.2 of the mean velocity in our simulation and keep the average injection rate of either individual atoms (one-atom case) or pairs of atoms (two-atom case) equal to $1/\tau$. This situation is called ‘random injection case’.

**Figure 5:**
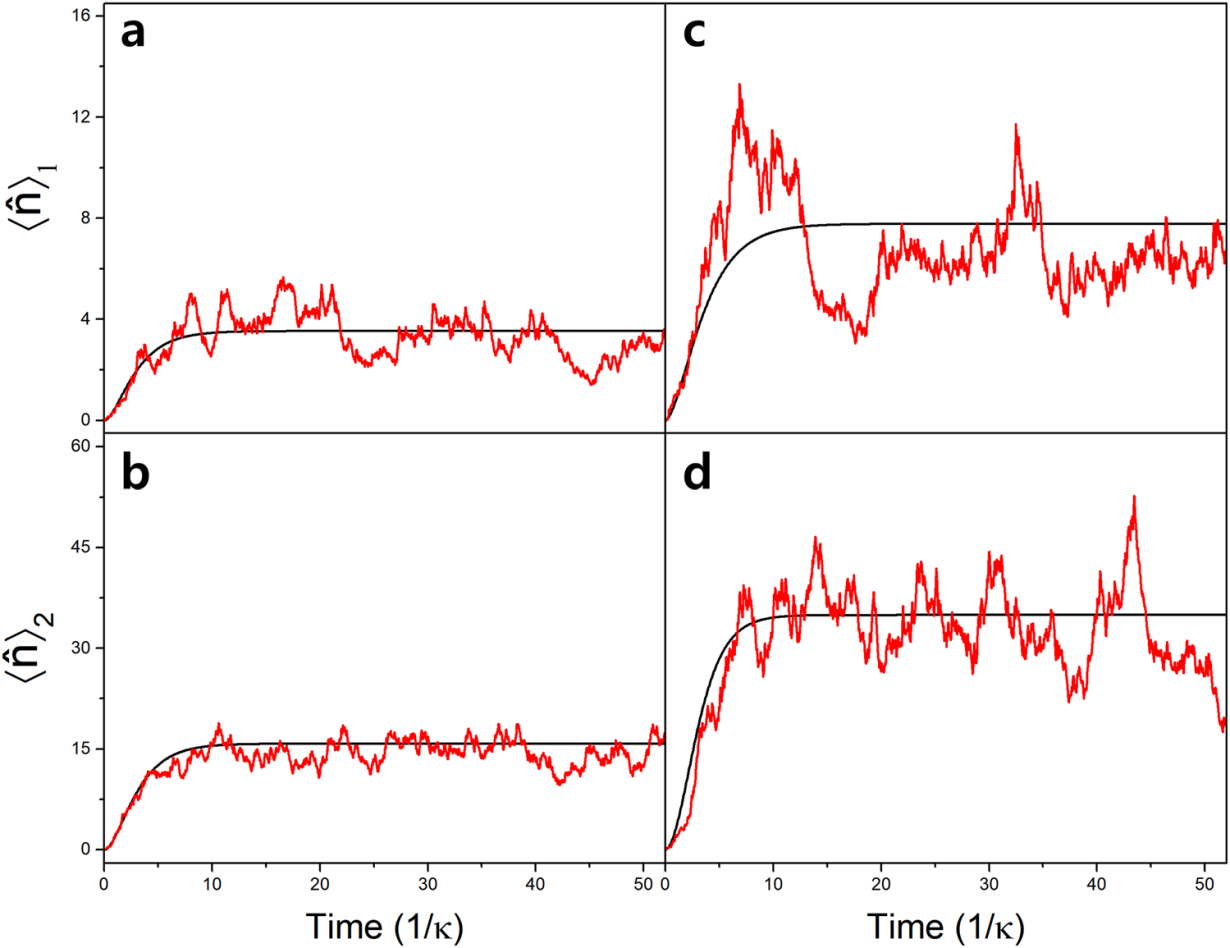
Time evolution of the average photon number inside the cavity. (**a,b**) One atom case **(a)** and two atoms case **(b)** with $g/\kappa =2$, $\Delta /\kappa =2$ and $\Omega /\kappa =10$. (**c,d**) One atom case **(c)** and two atoms case **(d)** with $g/\kappa =3$, $\Delta /\kappa =0$ and $\Omega /\kappa =15$. Black curves indicate the regular injection cases, and the red curves indicate the random injection cases. We assume the interaction time $\tau = 0.052/\kappa$, the cavity decay rate $1/\kappa = 1.93\mu$s and $\phi =\pi /2$. The unit time for the time scale is $1/\kappa = 1.93\mu$s.

In Fig. 5, the time evolution of the average photon number in the regular-injection case is compared with that of the random-injection case for a few sets of parameters. Due to the randomness, the random injection cases show fluctuating mean photon numbers, but their long-time averages exhibit a similar tendency of the mean photon numbers in the regular injection cases. The long-time average values in the random injection cases are compared with the expectation values of the photon number operator in the steady state in the regular injection cases in Table 1. These values are not much different, i.e., the random injection case does not deviate much from the regular injection case. This result suggests that the highly-idealized regular-injection case can be used as a guideline for designing actual experiments.

**Table 1:** Random vs. regular injection cases. The comparison of mean photon numbers in the random- and regular injection cases under two different conditions of Fig. 5. The percentage difference is defined as the quantity in the random injection divided by that in the regular injection minus 1.

		$\langle \hat{n} \rangle _1$	$\langle \hat{n} \rangle _2$	*R*
$g/\kappa =2, \Delta /\kappa =2,\Omega /\kappa =10$	Random injection	3.32	14.5	1.19
	Regular injection	3.54	15.8	1.23
(corresponding to Fig. 5a,b)	Difference (%)	$-6.36$	$-8.11$	$-3.38$
$g/\kappa =3,\Delta /\kappa =0,\Omega /\kappa =15$	Random injection	6.81	32.3	1.38
	Regular injection	7.78	35.0	1.25
(corresponding to Fig. 5c,d)	Difference (%)	$-12.6$	$-7.55$	$+10.3$

**Figure 6:**
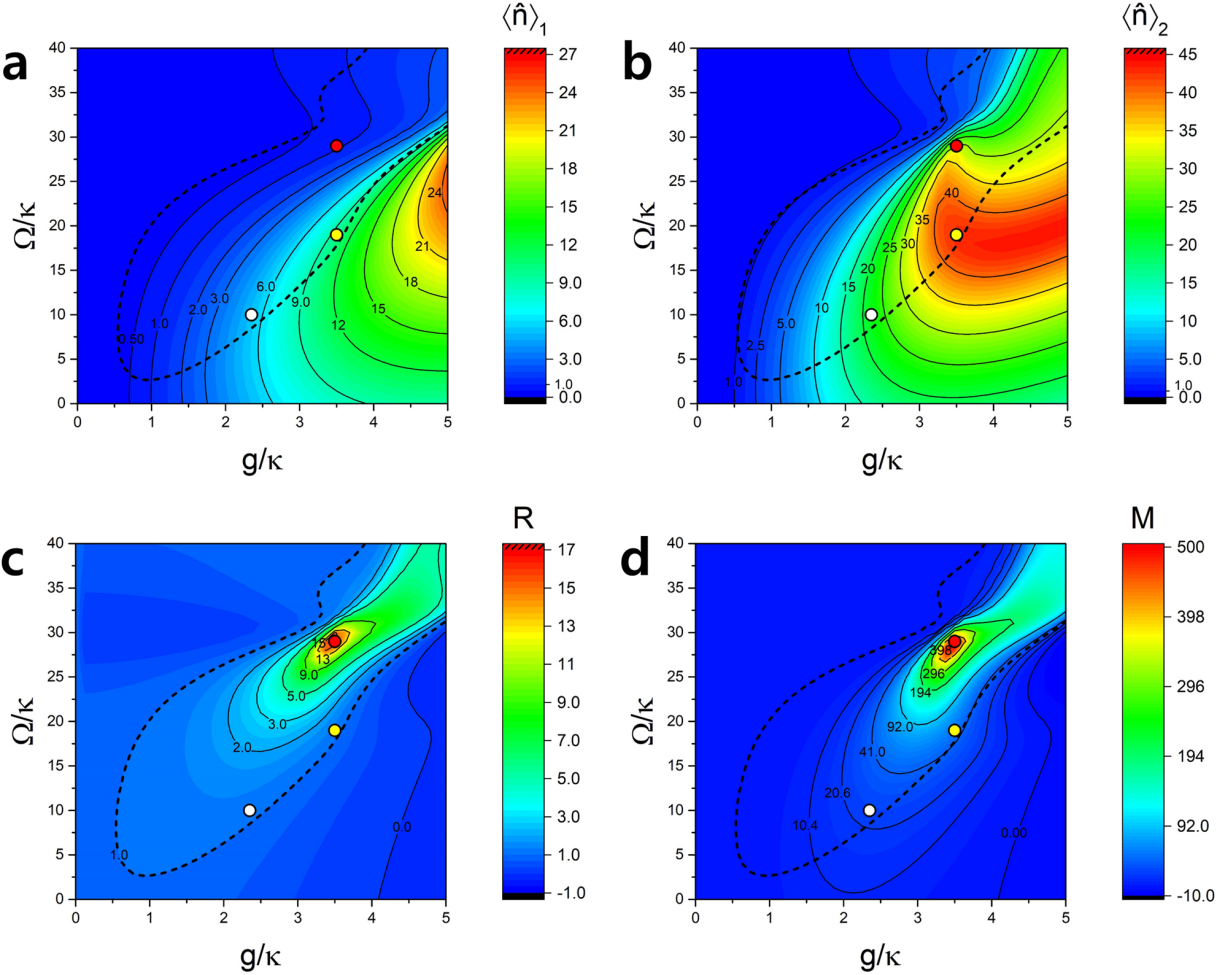
Magnified view of the low *g* region. (**a**) Average number of photons in the cavity for one-atom case. (**b**) The same for two-atom case. (**c**) Radiance witness *R*. Parameters are $\tau = 0.052/\kappa$, $1/\kappa = 1.93\mu$s and $\phi =\pi /2$. Hyperradiance region is inside the black dashed lines. We obtained the maximum mean photon number $\langle \hat{n} \rangle _2\simeq 43$ in two-atom case at around $\Omega /\kappa =19$ and $g/\kappa =3.5$ with $\langle \hat{n} \rangle _1=9.78$ and $R=1.19$ (indicated by yellow dots). The white dots correspond to $g/\kappa = 2.35$ (experimentally more easier to access) and $\Omega /\kappa =10$, resulting in $\langle \hat{n} \rangle _1= 5.19, \langle \hat{n} \rangle _2=22.0$ and $R=1.13$. The red dots are associated with the largest figure of merit $M=R\times \langle \hat{n} \rangle _2$ with $g/\kappa =3.5$ and $\Omega /\kappa =29$, giving rise to $\langle \hat{n} \rangle _2=30.7$ and $R=16.2$.

### Feasibility of experimental realization

Let us now examine the feasibility of experimental realization of the hyperradiance analyzed in the preceding sections. We first quote the experimental parameters in the coherent superradiance experiment reported in Ref.^26^. There we have $\tau =100$ ns, $g/2\pi =290$ kHz and $\kappa /2\pi =150$ kHz for a cavity with an 1.0 mm length and a finesse of $10^6$. The atomic transition is the $^1$S$_0\leftrightarrow ^3$P$_1$ transition of atomic barium at 791 nm with 50 kHz decay rate and the mean velocity is 755 m/s. These parameters give $g/\kappa \simeq$ 1.94 and $\kappa \tau \simeq$ 0.094.

The coupling constant *g* and the cavity decay rate $\kappa$ depend on the cavity length. The mode waist also depends on the length. In order to get $\kappa \tau \simeq$ 0.052, the same value as used in Figs. 2, 3, 4 and 5, we need a cavity length of 2.18mm, which gives the coupling constant $g/2\pi =163$ kHz, the cavity decay rate $\kappa /2\pi =68.8$ kHz, $\tau =120$ ns and thus $g/\kappa \simeq$ 2.35. This set of parameters is considered for a representative pump Rabi frequency $\Omega /\kappa =10$ in Fig. 6 (marked by white dots), corresponding to the low *g* region in the resonance case ($\Delta =0$). It gives a reasonably high mean photon number $\langle \hat{n} \rangle _2\simeq 22.0$ and a modest $R\simeq 1.13$.

The mean photon number can reach up to $\langle \hat{n} \rangle _2\simeq 43$ when $g/\kappa \simeq 3.5$ and $\Omega /\kappa =19$ (yellow dots). The necessary parameters for this can be obtained by choosing a cavity length of 1.0 mm, a radius of curvature of 5.0 cm for the mirrors and a finesse of 1,500,000 (the finesse in Refs.^26,28^ is $(1.0\sim 1.4)\times 10^6$). Since it is desirable to obtained both high *R* and $\langle \hat{n} \rangle _2$, we define a figure of merit *M* as $M\equiv R\times \langle \hat{n} \rangle _2$ and plot it in Fig. 6d. The largest *M* is obtained when $g/\kappa \simeq$3.5 and $\Omega /\kappa$=29 with $\langle \hat{n} \rangle _2\simeq 31$ and $R\simeq 16$(indicated by red dots). This corresponds to hyperradiance stronger than superradiance by 8.5 times. The parameters considered here are not difficult to achieve and thus the experimental realization is within reach of the current experimental capability. One can further optimize the cavity length, the mirror radius of curvature and the atomic velocity for both more increased radiance witness and mean photon numbers.

To summarize, we have theoretically shown that hyperradiance can occur in the setting of a microlaser where a stream of coherent superposition-state of atom pairs traverses a high-*Q* cavity. Numerical simulations as well as analytic calculations are employed to find that hyperradiance occurs when the reabsorption of the cavity field by individual atoms is maximized but suppressed by pairs of atoms. The radiation is greatly enhanced by introducing an additional pump field intersecting the cavity mode with a phase opposite to that of the atom-cavity coupling so that the atomic polarization is kept maximized during the atom-cavity interaction time. We also examine the feasibility of realizing hyperradiance in experiments. We find that random injection of atom pairs does not affect the result when time averages are considered and that a significant degree of hyperradiance can be observed with the experimental parameters reported in the literature. The present study can be utilized to achieve efficient light sources in the visible region, which would useful in areas like lighting applications^33^ and visible light communication^34^.

## Methods

### Derivation of Eqs. (5)–(7) by using semiclassical calculations

We follow the calculation procedures of Ref.^35^. First, we normalize the electric field as $\alpha$, the atomic polarization as $\sigma$ and the atomic population inversion as *r*. We can write a set of equations for these variables as follows.

13a $$\begin{aligned} \dot{\alpha }+\frac{\kappa }{2} \alpha +i\left( \omega _c-\omega _{p2}\right) \alpha\, = \, & {} -g\sigma \end{aligned}$$

13b $$\begin{aligned} \dot{\sigma }+\gamma \sigma +i\left( \omega _a-\omega _{p2}\right) \sigma\, = \, & {} -(g\alpha -\Omega ) r \end{aligned}$$

13c $$\begin{aligned} \dot{r}+2\gamma \left( r+1\right)\, = \, & {} 4\mathrm{Re}\left[ (g\alpha ^*-\Omega ^*)\sigma \right] , \end{aligned}$$

where $\sigma$ and *r* are the summations of individual atomic polarizations and population inversions, respectively, so $\sigma = \sum ^N_{i=1}\sigma _i$ and $r=\sum ^N_{i=1}r_i$ for *N* atoms in the cavity at the same time. The variables in the above equations are slowly varying variables with respect to the frequency $\omega _{p2}$. Now we apply the resonance condition $\omega _c=\omega _{p1}=\omega _{p2}\ne \omega _a$ and define the detuning as $\Delta =\omega _a-\omega _c$. Additionally, we assume that we can ignore the atomic decay rate $\gamma$ due to the very short interaction time $\tau$ such that $\gamma \tau \ll 1$. Under these conditions, the above equations become

14a $$\begin{aligned} \dot{\alpha }+\frac{\kappa }{2} \alpha\, = \, & {} -g\sigma \end{aligned}$$

14b $$\begin{aligned} \dot{\sigma }+i\Delta \sigma\, = \, & {} -(g\alpha -\Omega ) r \end{aligned}$$

14c $$\begin{aligned} \dot{r}\, = \, & {} 4\mathrm{Re}\left[ (g\alpha ^*-\Omega ^*)\sigma \right] , \end{aligned}$$

from which we can obtain the following relation.

15a $$\begin{aligned} \frac{d}{dt}\left( 4\sigma ^*\sigma +r^2\right)\, = \, & {} 0, \end{aligned}$$

15b $$\begin{aligned} \left( 4\sigma ^*\sigma +r^2\right)\, = \, & {} N^2 \end{aligned}$$

For the atomic initial state $\left( \vert \mathrm g \rangle +e^{-i\phi }\vert \mathrm e \rangle \right) /\sqrt{2}$ and the field initial state as a vacuum state $\vert 0 \rangle$, we obtain the initial values of those variables as $\sigma (0)=-\frac{N}{2}e^{i\left( -\phi +\frac{\pi }{2}\right) }$, $r\left( 0\right) =0$ and $\alpha (0)=\alpha _0$, where $\alpha _0$ is the semiclassical field strength right after the previous atom-cavity interaction. Using Eq. (15), we can write $\sigma \left( t\right)$ and $r\left( t\right)$ in terms of new variables $\beta (t)$ and $\theta (t)$.

16a $$\begin{aligned} \sigma (t)\, = \, & {} -\frac{N}{2}\cos {\theta (t)} e^{i\beta (t)} \end{aligned}$$

16b $$\begin{aligned} r(t)\, = \, & {} N\sin {\theta (t)}, \end{aligned}$$

with initial conditions $\beta (0)=-\phi +\frac{\pi }{2}$ and $\theta (0)=0$. Substituting Eqs. (16a) and  (16b) into Eq. (14b), We obtain following equation.

17 $$\begin{aligned} \dot{\theta } \sin {\theta }-i\dot{\beta }\cos {\theta }-i\Delta \cos {\theta }=-2\left( g\alpha -\Omega \right) \sin {\theta }e^{-i\beta } \end{aligned}$$

Using Eq. (17) and its complex conjugate form, we can derive the following equations.

18a $$\begin{aligned} \dot{\theta }\, = \, & {} \left( \Omega -g\alpha \right) e^{-i\beta }+\mathrm{c.c.} \end{aligned}$$

18b $$\begin{aligned} 2i\dot{\beta }\cos {\theta }+2i\Delta \cos {\theta }\, = \, & {} \left( g\alpha -\Omega \right) \sin {\theta }e^{-i\beta }-c.c. \end{aligned}$$

Since $\alpha , \theta$ and $\beta$ do not change much during the short interaction time $\tau$, we can substitute their initial values in Eq. (18). We then obtain the approximated time evolution of $\theta$ and $\beta$ as follows:

19a $$\begin{aligned} \theta (t)\approx & {} (\Omega -g\alpha _0)e^{i\left( \phi -\frac{\pi }{2}\right) }t+c.c. \end{aligned}$$

19b $$\begin{aligned} \beta (t)\approx & {} -\phi +\frac{\pi }{2}-\Delta t, \end{aligned}$$

for $0\le t\le \tau$. Since the interaction time is very short, we can consider the change of the field variable $\alpha$ as being continuous. In the steady state, $\alpha \simeq \alpha _0$, so $\alpha _0$ in Eq. (19a) can be replaced with $\alpha$. Substituting Eqs. (19) and  (16) into Eq. (14a) with expression of $\alpha$ as $\alpha =\sqrt{n}e^{i\xi }$. we obtain $\dot{n}$ and $\dot{\xi }$ equations by satisfying the real and imaginary parts of Eq. (14a). For a steady state solution of *n*, we require *n* to be slowly varying but $\dot{\xi }=0$, from which we obtain the steady-state phase of the cavity field $\xi _{\rm{st}}$ as

20 $$\begin{aligned} \xi _{\rm{st}}\approx -\phi +\frac{\pi }{2}-\Delta \tau , \end{aligned}$$

where $\tau$ is the atom-cavity interaction time. In the remaining $\dot{n}$ equation, $\xi (t)$ is replaced with $\xi _{\rm{st}}$ and the time dependence between time 0 and $\tau$ in the equation is averaged out by introducing the following integral

21 $$\begin{aligned} n\equiv \frac{1}{\tau }\int ^{\tau }_0 n(t')dt'. \end{aligned}$$

The result is

22 $$\begin{aligned} \dot{n}+\kappa n\, = \, & {} \frac{Ng\sqrt{n}}{2\tau }\frac{\sin {2(g\sqrt{n}\cos {\Delta \tau }+\mathfrak {I}[\Omega e^{-i\phi }])\tau }+\sin {\Delta \tau }}{\Delta +2g\sqrt{n}\cos {\Delta \tau }+2\mathfrak {I}[\Omega e^{-i\phi }]} \nonumber \\&+(\Delta \longleftrightarrow -\Delta ). \end{aligned}$$

We now calculate the steady state solution ($\dot{n}=0$) on resonance ($\Delta =0$). We assume the initial state of atom as $\frac{1}{\sqrt{2}}\left( \vert \mathrm g \rangle -i\vert \mathrm e \rangle \right)$ so that $\phi =\frac{\pi }{2}$ and we can treat $\Omega$ as a real number without loss of generality. We also assume both coupling *g* and Rabi frequency $\Omega$ are not so large that they satisfy $g\sqrt{n}\tau ,\Omega \tau \ll 1$. Then, we can expand sine and cosine functions to the order of $O\left( \tau ^3\right)$—hyperradiance is not seen up to the 2nd order and it appears only when we expand the terms to the 3rd order—and obtain a solvable second-order equation for $\sqrt{n}$. The result is

23a $$\begin{aligned} \kappa n\, = \, & {} N g\sqrt{n}\frac{\sin {2\left( g\sqrt{n}-\Omega \right) \tau }}{2\left( g\sqrt{n}-\Omega \right) \tau } \end{aligned}$$

23b $$\begin{aligned}\approx & {} N g\sqrt{n}\left\{ 1-\frac{1}{3!}\left[ 2\left( g\sqrt{n}-\Omega \right) \tau \right] ^2\right\} \end{aligned}$$

Equation (23b) is a quadratic equation for $\sqrt{n}$. The solution is

24 $$\begin{aligned} n\approx & {} \left[ \frac{-3+4N (\Omega /\kappa ) (g\tau )^2+\sqrt{9-24N (\Omega /\kappa )(g\tau )^2+24N^2 (g/\kappa )^2(g\tau )^2}}{4N(g/\kappa ) (g\tau )^2}\right] ^2 \nonumber \\\approx & {} \left( \frac{gN}{\kappa }\right) ^2\left[ 1 -\frac{2}{3} (\Omega \tau )^2+\frac{4}{3}N ( \Omega /\kappa )(g\tau )^2 -\frac{2}{3}N^2 (g/\kappa )^2 (g\tau )^2\right] ^2. \end{aligned}$$

In order to obtain the condition for the maximum radiance witness *R*, we differentiate Eq. (24) with respect to $\left( g\tau \right)$ with $\Omega /\kappa$ fixed. We denote *n* for $N=1$ and $N=2$ as $n_1$ and $n_2$, respectively. From $\partial R/\partial (g\tau )=0$, we can obtain the condition for maximizing *R* as

25 $$\begin{aligned} \Omega \tau =\frac{3\left( g\tau \right) ^2}{\kappa \tau }, \end{aligned}$$

which can be rewritten as

26 $$\begin{aligned} \Omega /\kappa = 3(g/\kappa )^2. \end{aligned}$$

Another partial differential $\partial R/\partial (\Omega \tau )$ produces the condition for minimizing *R*, which has little significance to us and is thus not explicated here.

### Detailed consideration of the two-atom full Rabi frequencies

Consider two atoms interacting with a very weak cavity field. The Hamiltonian is given by

27 $$\begin{aligned} H=\frac{\hbar \Delta }{2}\sigma _{z1}+\frac{\hbar \Delta }{2}\sigma _{z2}+\hbar g\left( a\sigma ^+_1+a^\dagger \sigma ^-_1\right) +\hbar g\left( a\sigma ^+_2+a^\dagger \sigma ^-_2\right) \end{aligned}$$

where we keep the interaction between the atoms and the cavity field and neglect the interaction with the pump $\left( \Omega \ll \Delta , g\right)$. We consider only the triplet states $\vert 1,1 \rangle =\vert \mathrm{ee},n \rangle$, $\vert 1,0 \rangle =\left( \vert \mathrm{eg},n+1 \rangle +\vert \mathrm{ge},n+1 \rangle \right) /\sqrt{2}$ and $\vert 1,-1 \rangle =\vert \mathrm{gg},n+2 \rangle$ coupled to the cavity field and exclude the singlet state $\vert 0,0 \rangle =\left( \vert \mathrm{eg},n+1 \rangle -\vert \mathrm{ge},n+1 \rangle \right) /\sqrt{2}$ because the singlet state is uncoupled from both the triplet states and the cavity field. Then, we can represent the wave function $\vert \Psi \rangle =c_1\vert 1,1 \rangle +c_0\vert 1,0 \rangle +c_{-1}\vert 1,-1 \rangle$ as a three-dimensional vector

28 $$\begin{aligned} \vert \Psi \rangle =\begin{pmatrix} c_1\\ c_0\\ c_{-1} \end{pmatrix} \end{aligned}$$

and the Hamiltonian as a three-by-three matrix.

29 $$\begin{aligned} H/\hbar =\begin{pmatrix} \Delta &{} \sqrt{2}g\sqrt{n+1} &{} 0\\ \sqrt{2}g\sqrt{n+1} &{} 0 &{} \sqrt{2}g\sqrt{n+2}\\ 0 &{} \sqrt{2}g\sqrt{n+2} &{} -\Delta \end{pmatrix} \end{aligned}$$

Solving the eigenvalue equation, we obtain a cubic equation

30 $$\begin{aligned} \lambda ^3-\{\Delta ^2+2g^2\left( 2n+3\right) \}\lambda +2\Delta g^2=0. \end{aligned}$$

The solutions $\lambda _1$, $\lambda _2$ and $\lambda _3$ correspond to the full Rabi frequencies of the two-atom case. The exact form of $\lambda _1$, $\lambda _2$ and $\lambda _3$ can be obtained by applying Cardano’s formula. The resulting form is not reproduced here because it is complicated, and thus it is obviously very distinct from the form of the full Rabi frequency of the one-atom case.

## Data Availability

The datasets generated during the current study are available from the corresponding author on reasonable request.

## Code availability

The code that supports the findings of this study are available from the corresponding author upon reasonable request.
